# Final report of the SIOP-CNS-GCT II protocol for germinoma: Omitting radiotherapy boost is safe in localized disease in complete remission after chemotherapy

**DOI:** 10.1093/neuonc/noag127

**Published:** 2026-06-03

**Authors:** Gabriele Calaminus, Claire Alapetite, Rolf D Kortmann, Thankamma Ajithkumar, Martin Zimmermann, Hervé J Brisse, Maria Luisa Garrè, Giovanni Morana, Thomas Czech, Andreas Peyrl, Nicolas U Gerber, Stefan Schönberger, Martin Heimbrodt, Celestina Saager, Yannik Rambau, Brigitte Bison, Didier Frappaz, Cecile Faure-Conter, Matthew J Murray, James C Nicholson

**Affiliations:** Department of Pediatric Hematology and Oncology, University Hospital Bonn, Bonn, Germany; Department of Radiation Oncology and Proton Center, Institute Curie, Paris, France; Department of Radiotherapy and Radio-Oncology, University Hospital Leipzig, Leipzig, Germany; Department of Clinical Oncology, Cambridge University Hospitals NHS Foundation Trust, Cambridge, UK; Department of Pediatric Hematology and Oncology, Hannover Medical High School, University Hospital Hannover, Hannover, Germany; Department of Imaging, Institut Curie, Paris, France; Neuro-Oncology Unit, IRCCS Giannina Gaslini, Genova, Italy; Department of Neurosciences, Neuroradiology Unit, University of Turin, Turin, Italy; Department of Neurosurgery, Medial University Vienna, Vienna, Austria; Department of Pediatrics and Adolescent Medicine, Medical University Vienna, Vienna, Austria; Department of Oncology, University Children’s Hospital, Zurich, Switzerland; Department of Pediatric Hematology and Oncology, Clinic of Pediatrics III, University Hospital of Essen, University of Duisburg-Essen, Essen, Germany; Department of Pediatric Hematology and Oncology, University Hospital Bonn, Bonn, Germany; Department of Pediatric Hematology and Oncology, University Hospital Bonn, Bonn, Germany; Department of Pediatric Hematology and Oncology, University Hospital Bonn, Bonn, Germany; Diagnostic and Interventional Neuroradiology, Neuroradiological Reference Center for the HIT-Studies of the GPOH, Faculty of Medicine, University Augsburg, Augsburg, Germany; Institute of Pediatric Hematology and Oncology, Lyon, France; Institute of Pediatric Hematology and Oncology, Lyon, France; Department of Pathology, University of Cambridge, Cambridge, UK; Department of Pediatric Hematology and Oncology, Cambridge University Hospitals NHS Foundation Trust, Cambridge, UK; Department of Pediatric Hematology and Oncology, Cambridge University Hospitals NHS Foundation Trust, Cambridge, UK; Department of Pediatrics, University of Cambridge, Cambridge University Hospitals NHS Foundation Trust, Cambridge, UK

**Keywords:** event-free survival, intracranial germinoma, overall survival, risk-adapted therapy, whole-ventricular radiotherapy

## Abstract

**Background:**

For localized germinoma, whole-ventricular radiotherapy (WVRT) is standard-of-care in Europe; 24 Gray (Gy) with 16 Gy boost following chemotherapy. The SIOP-CNS-GCT-II trial aimed to examine the effect of response-adapted radiotherapy on patient outcome.

**Methods:**

Patients with localized germinoma received 4 courses of “carboPEI” chemotherapy, then 24 Gy WVRT if in complete remission (CR), with an additional 16 Gy tumor boost if residual disease was present. Metastatic cases received 24 Gy craniospinal radiotherapy (CSRT) with 16 Gy boost to all sites.

**Results:**

Between 2012 and 2018, 227 fully-staged germinoma patients were treated according to protocol. Five-year event-free (EFS) and overall survival (OS) for 166 localized germinoma were 0.94 ± 0.02 and 0.98 ± 0.01, respectively. Sixty-five of 166 (39.2%) were in CR after chemotherapy, of whom 64/65 received 24 Gy WVRT, only 2 of whom (2/64; 3.1%) relapsed. Of 90/166 patients in partial remission (PR) after chemotherapy, 88/90 received 24 Gy WVRT + 16 Gy; 2 relapsed (2/88; 2.3%). Of the 11 remaining patients, 8 had stable disease (SD) after chemotherapy; 7/8 received 24 Gy WVRT + 16 Gy boost and 1 received 24 Gy WVRT + 30 Gy boost because of a teratoma component; none relapsed. Three remaining patients with progressive disease (PD) during chemotherapy had variable treatments, due to differing histologies and disease spread; 1 relapsed. Five-year EFS and OS for 61 metastatic germinoma were 0.98 ± 0.02 and 1.00 ± 0.00, respectively, with 55/61 (90.2%) in CR and 5/61 (11.5%) in PR after 24 Gy CSRT + 16 Gy; 1 relapsed.

**Conclusions:**

Response-adapted radiotherapy for localized germinoma led to excellent survival outcomes. Further treatment de-escalation should be considered in future strategies to reduce treatment burden and late effects.

Key Points

- Patients with CNS germinoma have excellent outcomes; focus is on reducing late effects
- In this trial, 5-year event-free survival was 94±2% in localized patients in complete remission after chemotherapy, who then received 24 Gy WVRT without boost

Importance of the Study

The SIOP-CNS-GCT-II trial confirms that, for localized, including bifocal, germinoma patients who are in complete remission (CR) after induction chemotherapy, whole ventricular radiotherapy (WVRT) with 24 Gy without tumor bed boost is sufficient for disease control and can now be defined as the gold standard in Europe for this disease group. For patients with metastatic germinoma, 24 Gy craniospinal radiotherapy (CSRT) with a 16 Gy tumor bed boost without chemotherapy effectively controls disseminated disease. Given excellent outcomes, future studies should investigate possibilities for decreasing overall chemotherapy burden for patients with localized disease, as well as radiotherapy dose reductions. For metastatic germinoma, the use of chemotherapy may also allow radiotherapy dose reductions to the primary tumor and metastases. Such approaches will reduce the burden of treatment-associated late effects for this cohort.

Intracranial germ cell tumors (GCT) constitute 3-4% of primary central nervous system (CNS) tumors in the US and Europe. Management currently depends on the historical categorization into pure germinoma and non-germinomatous GCTs (NGGCTs).^1^ The latter group includes all those with 1 or more malignant NGGCT components, alone or in combination with germinoma or teratoma. Patients with pure germinoma (subsequently referred to only as “germinoma”) have tumors comprising only germinoma as the malignant component (± teratoma). Germinomas are exceptionally chemotherapy and radiotherapy (RT) sensitive and have superior outcomes, in terms of both event-free (EFS) and overall (OS) survival, compared with those patients with NGGCTs.^1^

Large retrospective series and prospective trials have shown that craniospinal RT (CSRT) alone is an effective therapy, yielding high cure rates for CNS germinoma.^2^^,^^3^ However, long-term survivors, particularly children and pre-pubertal adolescents, are at risk of substantial late sequelae of RT.^4^ Attempts have therefore been made to limit RT treatment volumes and/or doses to diminish the potential adverse effects on the CNS, endocrine function, and spinal growth.^5-7^ A dose of 40 Gy to the primary tumor site (focal RT) achieved high local control rates. However, exclusively focal RT, when delivered in combination with chemotherapy, resulted in an increased risk of ventricular relapses.^8^^,^^9^ Chemotherapy-only approaches are also unsatisfactory, with cure rates of only ∼50%; thus delivery of RT is essential for optimal disease control.^10-12^ RT alone with smaller treatment volumes (such as omission of spinal RT for localized cases) was effective,^13^^,^^14^ and the use of chemotherapy allowed for further reductions in RT doses and/or volumes,^15^ with evidence of relatively minimal neuropsychological morbidity over time.^16^ Consequently, whole ventricular RT (WVRT) was identified as the optimal and safest treatment to control such subclinical ventricular disease. In conjunction with chemotherapy, a dose reduction to 24 Gy to the whole ventricle seemed feasible based on Japanese studies, which found an 86-100% relapse-free survival rate at 5 years.^17^^,^^18^

The multinational European SIOP-CNS-GCT-II trial was a prospective, non-randomized trial for the diagnosis and treatment of children, adolescents, and adults with CNS GCTs. The basis of treatment for localized germinoma was to maintain the chemotherapy administered in the combined treatment arm of the prior European study SIOP-CNS-GCT-96^2^, but to deliver WVRT as standard-of-care to reduce the risk of ventricular relapses. In this context, the trial aimed to use WVRT alone for patients in CR after chemotherapy to reduce the total tumor bed dose from 40 to 24 Gy, limiting long-term effects of therapy.

## Methods

### Trial Details

The European SIOP-CNS-GCT-II trial (EudraCT number: 2009-018072-33) was sponsored by the University Hospital Münster, Germany; study number: UKM08_0057. The date of trial authorization, after ethical approval by the University of Münster, was 15/06/2011 (Germany, Federal Institute for Drugs and Medical Devices [BfArM]). The first patient was recruited on 02/02/2012, and the registration lock was on 01/07/2018. Data were collected using an electronic data capture system (MARVIN, XClinical, Munich, Germany). Data quality was checked within the MARVIN Remote Data Entry (RDE) system by the data management team from the trial coordination data center on a regular basis. Recruitment evaluation and toxicity analyses were performed annually by the Safety Desk (Sponsor: University Hospital Münster, Germany).

### Primary Trial Objectives

These were to:

- Maintain current EFS rates (>80%)^17^^,^^18^ using a risk-adapted approach;
- In localized germinoma [including bifocal (see definition below)]: to omit CSRT by using combined treatment with standard chemotherapy and ventricular radiotherapy ± tumor bed boost;
- In metastatic disease: to maintain current EFS (>90%) using CSRT.

### Diagnostic and Staging Procedures

**Figure 1.**
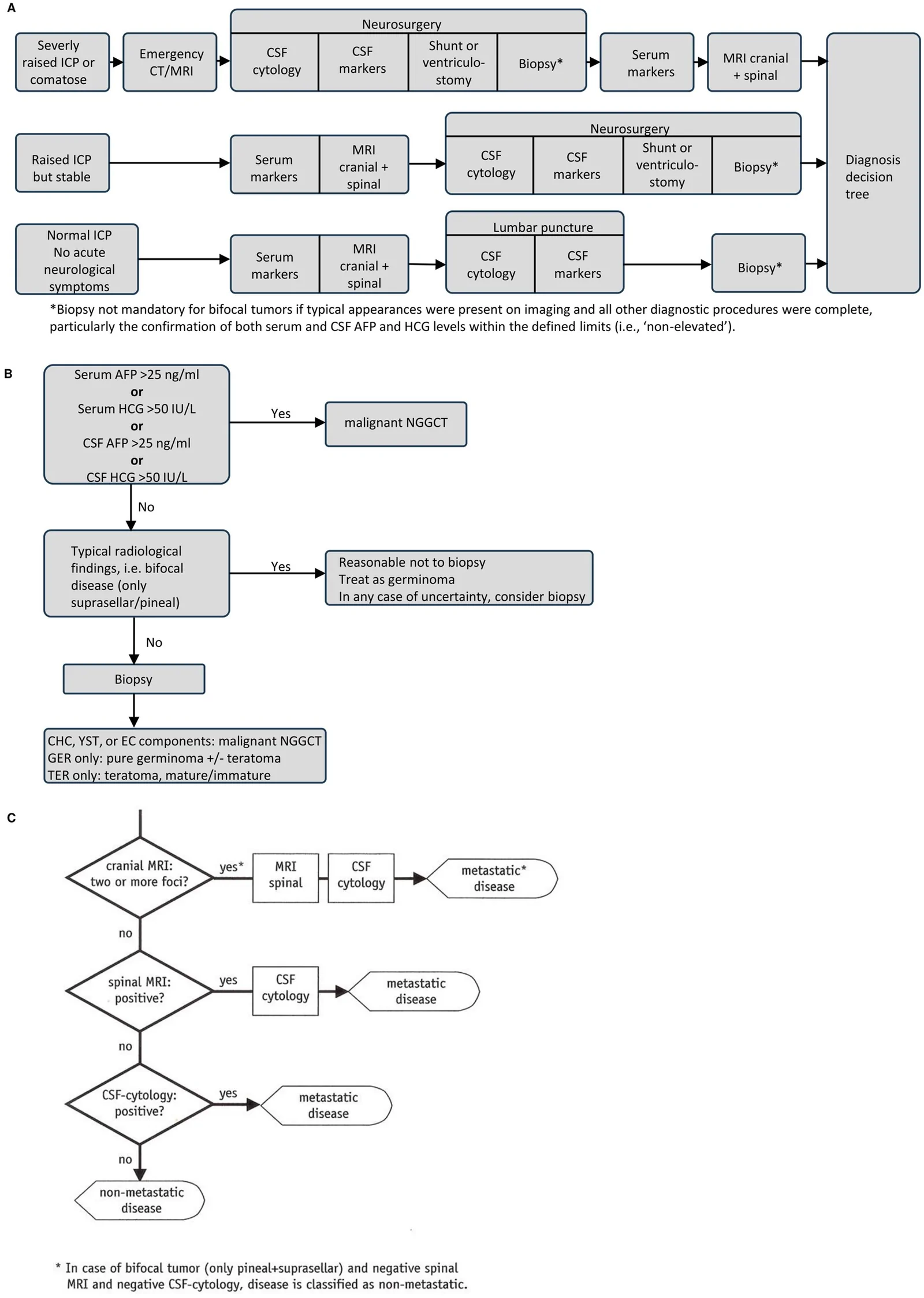
Diagnostic work-up and staging for presumed intracranial germ cell tumor (GCT) patients as per the SIOP-CNS-GCT-II trial protocol. (A) Initial management for patients with a suspected intracranial GCT based on the presence or absence of raised intracranial pressure. Key: ICP: intracranial pressure; CT: computed tomogram; MRI: magnetic resonance imaging; CSF: cerebrospinal fluid. (B) Diagnosis decision tree for patients with an intracranial GCT. Key: AFP: alpha-fetoprotein; HCG: human chorionic gonadotropin; DI: diabetes insipidus; GER: pure germinoma; CHC: choriocarcinoma; YST: yolk sac tumor (endodermal sinus tumor); EC: embryonal carcinoma; TER: teratoma; NGGCT: non-germinomatous germ cell tumor. (C) Staging for dissemination using full neuroaxis imaging and CSF cytology.

Patients with germinoma were diagnosed based on biopsy, performed in the context of tumor marker levels of alpha-fetoprotein (AFP) and human chorionic gonadotropin (HCG), in both serum and cerebrospinal fluid (CSF), being below defined European thresholds [ie, AFP 25 ng/ml (20.8 kU/l); HCG 50 IU/l] (Figure 1A-C). Bifocal tumors (pineal and suprasellar site only; no other disease elsewhere) were considered non-metastatic and were treated in the same way as unifocal, localized tumors. Of note, for the SIOP-CNS-GCT-II study, patients with a pineal tumor and diabetes insipidus (DI), who did not have macroscopic suprasellar disease on imaging, were not considered bifocal. In patients with bifocal tumors, a European consensus was reached that a biopsy for histological verification was not mandatory if typical appearances were present on imaging and all other diagnostic procedures were complete, particularly the confirmation of both serum and CSF AFP and HCG levels within the defined limits (ie, “non-elevated”). Radiologically, typical germinoma appearances included homogeneous and well-defined enhancing lesion(s), and occasional hemorrhage or calcifications.^19^ Other than bifocal cases, those with non-contiguous disease on full neuroaxis imaging and/or positive CSF cytology were defined as metastatic disease (Figure 1A-C). Information regarding the source of CSF for marker estimation and cytological assessment (ventricular versus lumbar) was not routinely collected during the trial. Patients with incomplete staging were excluded from further analysis.

### Cohort Description

**Figure 2.**
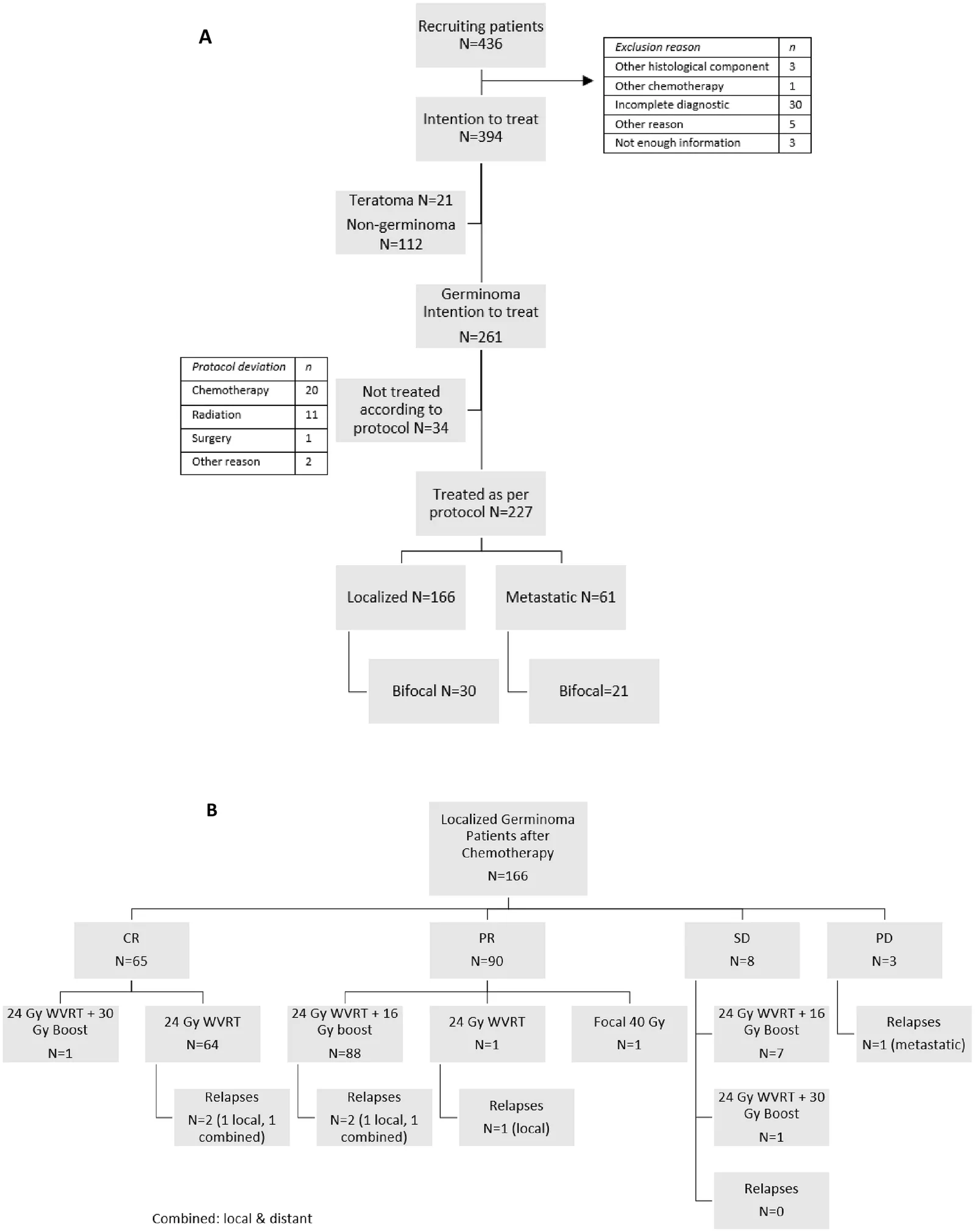
CONSORT diagram for patients with intracranial germinoma on the SIOP-CNS-GCT-II trial.

Between 01/02/2012 and 01/07/2018, 436 patients with CNS GCTs were enrolled in the SIOP-CNS-GCT-II trial, from Austria (*n* = 9), France (*n* = 128), Germany (*n* = 164), Italy (*n* = 3), Norway (*n* = 8), Sweden (*n* = 19), Switzerland (*n* = 11), and the UK (*n* = 94). Following SIOP-CNS-GCT-II protocol diagnostic work-up and staging (Figure 1B), eligibility criteria were not met in 42/436 (9.6%) patients, leaving 394 (90.4%) patients for per-protocol analysis (Figure 2A). Of these 394, 261 (66.2%) patients had germinoma, 112 (28.4%) NGGCT, and 21 (5.3%) pure teratoma. Of those with appropriate work-up/staging, 227/261 (87.0%) patients with germinoma were treated as per trial protocol (Figure 2A). The results for patients with NGGCTs and a more detailed analysis of germinomatous bifocal tumors will be published separately.

### Initial Surgery and Pathological Analysis

The diagnosis of intracranial germinoma was histologically confirmed by local pathologists in 208/227 patients recruited to the trial and treated as per protocol; in 19/30 cases with a bifocal tumor, the diagnosis was made by typical imaging and tumor markers alone, as defined in the study protocol. Histologically, tumors were classified according to the World Health Organization diagnostic criteria^20^ as germinoma in 194/208 (93.3%) of cases. The remaining 14/208 (6.7%) of patients also had additional teratoma components present, with mature (*n* = 12), immature (*n* = 1), or combined (mature and immature; *n* = 1) elements described. In the protocol, the presence of teratoma, mature or immature, with germinoma at diagnosis did not alter initial management nor result in re-classification as an NGGCT. In 2 cases, the histology reports described HCG-positive syncytiotrophoblastic giant cells, as can be observed in germinoma.^1^ Central pathological review was performed as required on a national basis.

### Treatment

#### Localized germinoma

**Figure 3.**
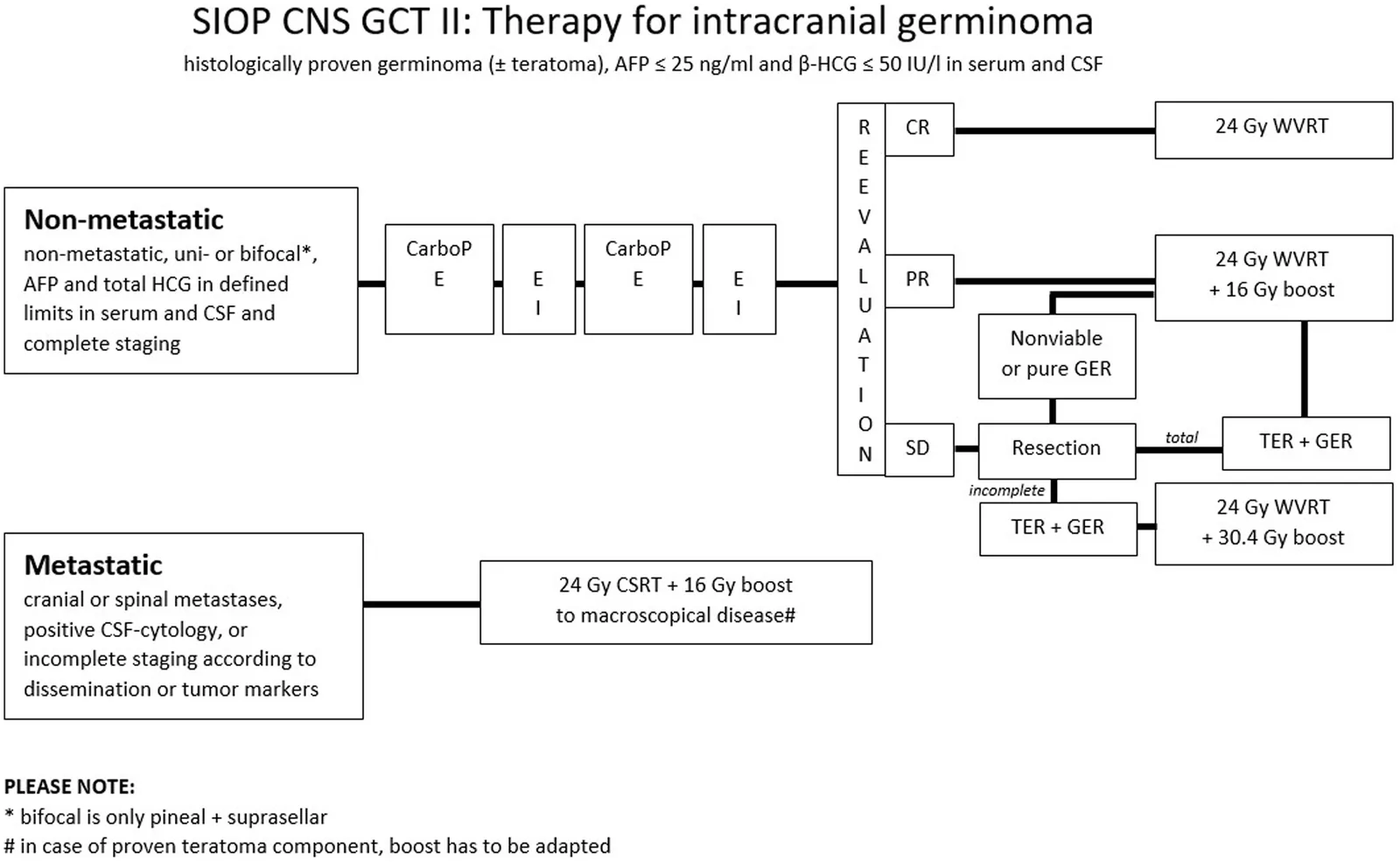
Treatment outline for patients with intracranial germinoma on the European SIOP-CNS-GCT-II trial. Key: AFP: alpha-fetoprotein; EVAL: evaluation (MRI/markers); CR: complete remission; PR: partial remission; SD: stable disease; TU: tumour bed; WVRT: whole ventricular radiotherapy; CSRT: cranio-spinal radiotherapy; GER: germinoma; TER: teratoma; GCT: germ cell tumor.

In the trial, a risk-adapted approach was used to maintain the current EFS. “CarboPEI” chemotherapy, comprising 2 courses of carboplatin/etoposide (600 mg/m^2^/day1 and 100 mg/m^2^/day1-3, respectively) alternating with 2 of ifosfamide/etoposide (1800 m^2^/day1-5 and 100 mg/m^2^/day1-3, respectively), was commenced as soon as possible following diagnosis (Figure 3), although no specific time limit from diagnosis to commencing therapy was stipulated in the trial protocol. Evaluation of chemotherapy response was confirmed by national reference radiologists (central review) in real-time, according to the neuroradiology guidelines in the trial protocol, to determine CR, partial remission (PR), stable disease (SD), or progressive disease (PD). All unclear cases were discussed with the international trial panel, which was composed of all national reference staff. Tumor markers did not routinely factor into response assessments, as germinoma typically present without elevated markers, and by trial definition, below specific thresholds.

Patients in CR following chemotherapy received 24 Gy WVRT [proton beam therapy or photon intensity-modulated radiation therapy (IMRT) technique], including coverage of the original tumor bed and the lateral, third, and fourth ventricles in this target volume. Biopsy tracts and the pre-pontine cistern were not part of the elective volume. A CTV margin of 5 mm was added to the ventricular system and the tumor bed. In this group, the 16 Gy boost to the tumor bed area was not delivered.

Patients with PR or SD following chemotherapy received 24 Gy WVRT, ensuring that the original tumor bed and any residual disease were included in the target volume, followed by a 16 Gy boost to the primary tumor bed (Figure 3). The ventricular delineation included the wall of the distal part of the horns in the lateral ventricle, the third ventricle, including all recesses and specifically the infundibular recess, fourth ventricle, foramen of Monroe, sylvian aqueduct, foramen of Luschka, and inferior limit to the occipital hole.^21^ In 8 patients with SD, second-look surgery before the start of radiotherapy was recommended by the trial protocol. However, in the rare situation of SD where resection was attempted but incomplete, and histology showed a teratoma component, a 30.4 Gy tumor bed-boost was delivered, resulting in a 54.4 Gy total dose (Figure 3), similarly to NGGCT cases.^22^  *Metastatic Germinoma*: All patients received 24 Gy CSRT with 16 Gy boost to the tumor bed and all sites of macroscopic metastatic disease, intracranial and/or spinal. Tumor markers were not part of the routine response assessments in either localized or metastatic germinoma cases.

#### Handling of teratoma components

The rationale for a higher radiotherapy boost in teratoma is the effect of radiotherapy on proliferating basal epithelial cells, preventing cyst growth. The exact dose required in this rare situation is unknown, but patients are often treated with the same dose as craniopharyngioma, based on histological similarity, hence the dose of 50-54 Gy in 25-30 fractions.^23^^,^^24^

### Follow-up

Full clinical examination, including neurological status, vision, hearing, endocrine, and quality-of-life (QoL) assessments, together with laboratory studies including measurements of serum AFP/HCG, and MRI, were performed as per trial protocol in follow-up. MRI brain (±spine) and tumor markers were performed 6-monthly for the first 2 years of follow-up and annually thereafter for up to 5 years. In follow-up, localized cases alternated between brain and brain+spine MRI; metastatic cases had brain+spine MRI at each evaluation. At 5 years, brain MR angiography (MRA) was also recommended to look for any evidence of arteriopathy following radiotherapy.^18^ Long-term assessments included clinical status, relapse, second malignancy, and persistent side-effects of therapy. Taking into account the maximum follow-up time period of 5 years, the latest follow-up for a patient was 13/06/2023.

### Statistical Analysis

The probability of survival was estimated according to the Kaplan-Meier method. EFS was calculated to measure the proportion of patients who remained free of an event (relapse or death of any cause). Five-year OS was calculated from the date of diagnosis to the date of last follow-up or death. To compare the survival distributions of 2 groups, the log-rank test was used. With multivariate analysis, the variable responses after chemotherapy (CR/PR/SD/PD), biological sex, primary tumor site (pineal/suprasellar/bifocal/other), and metastatic status (localized/metastatic) within 5 years from diagnosis were examined. Data were recorded and monitored at the University Hospital Münster database. SAS (version 9.2 for Windows; SAS Institute, Cary, NC, US) was used for statistical analysis in University Hospital Hannover, Germany, by Dr. Martin Zimmermann. Toxicity was documented according to Common Terminology Criteria for Adverse Events (CTCAE) version 3.0. Significance for comparisons between groups was undertaken using the log-rank test with *P-*value <.05 considered significant.

## Results

### Demographic, Tumor Staging, and Tumor Site Information

Between 2012 and 2018, 227 patients with fully-staged germinoma were registered (166 localized; 61 metastatic). Notably, 56 of 227 patients (24.7%) were over the age of 18 years (y) at trial enrollment. At the final data-lock of June 13, 2023, median follow-up for the whole cohort was 5 y (range 0.5-5.0 y). Of the 227, patient age range was 5.0-41.0 y (median 14.1 y); 180 (79.3%) were male and 47 (20.7%) female. Staging identified that 166 (73.1%) cases were localized and 61 (26.9%) metastatic (Figure 2A). Of the 166 patients with localized germinoma, 91 (54.8%) had pineal disease, 44 (26.5%) suprasellar, 30 (18.1%) bifocal, and 1 (0.6%) disease at another site (hypothalamus). None of the 166 patients had a CSF AFP higher than 10 ng/ml. Only 2/166 localized germinoma patients had AFP serum levels between 10 and 25 ng/ml. One of these patients relapsed (patient 6384). Further details on this patient can be seen in Table 1. For the 61 patients with metastatic disease, 21 (34.4%) had a pineal primary tumor, 14 (23.0%) suprasellar, 21 (34.4%) bifocal, and 5 (8.2%) at other sites.

**Table 1.** Characteristics of patients with a relapse event on the SIOP-CNS-GCT-II trial

	Diagnosis group	**Localized**						Metastatic
	Patient	6176	6236	6148	6162	6233	6384	6149
	Sex	male	female	male	female	male	male	male
Primary diagnosis	Age (years)	14	10	9	12	14	14	10
	Tumor site	bifocal	suprasellar	pineal	bifocal	bifocal	pineal	pineal
	Metastases	No	No	No	No	No	No	Yes
	AFP serum (ng/ml)	7	2.2	1.6	4	3.37	24.1	1.21
	AFP CSF (ng/ml)	1	0.2	0.5	–	Normal range	1	1.21
	HCG Serum (IU/l)	1.4	3	3	2.9	4.2	1	1
	HCG CSF (IU/l)	5.6	3	5	–	24.2	2	1.2
	Status after Chemo	CR	CR	PR	PR	PR	PD	–
	Radiotherapy	Both sites; 24 Gy WVRT	24 Gy WVRT	24 Gy WVRT + 16 Gy boost	Both sites; 24 Gy WVRT + 16 Gy boost	Both sites; 24 Gy WVRT	24 Gy CSRT + 30 Gy boost	24 Gy CSRT + 16 Gy boost
	Status after RT	CR	CR	PR	PR	CR	PR	CR
At relapse	Time to relapse from original diagnosis (years; months)	0; 6	1; 5	2; 7	1; 9	4; 6	0; 9	0; 9
	Local/metastatic	Combined	Local	Local	Combined	Local	Metastatic	Metastatic
	Localization	CNS	CNS	CNS	CNS	CNS	CNS	Abdomen (patient had an abd. VP shunt)
	Within the irradiation field	Yes and no	Yes	Yes	Yes and no	Unknown	Yes	No
	Specified	Cerebellar above fourth ventricle and lumbosacral	Suprasellar and hypo-thalamus	Pituitary stalk	Pituary stalk and pineal and spinal	Infundi-bulum	Ventricle and meningeal seeding	Peritoneal metastasis
	Tumor components	Germinoma	Germinoma, chorio-carcinoma	Germinoma	Germinoma, choriocarcinoma	Germi-noma	Unknown	Germinoma
	Therapy	HIT-REZ protocol	CURE PEI and proton therapy	PEI-PEI-HD VP-TTP	PEI/HD PEI and CSRT	HIT REZ protocol	GemPox	JEB x6
After relapse	Outcome	Second relapse -> death of disease (DOD)	CR	PR	CR	Second relapse	CR	CR

Information with respect to age, site, dissemination, tumor markers, radiological response, time to relapse, site of relapse, and outcome patient status is listed.

### Localized Germinoma

**Figure 4.**
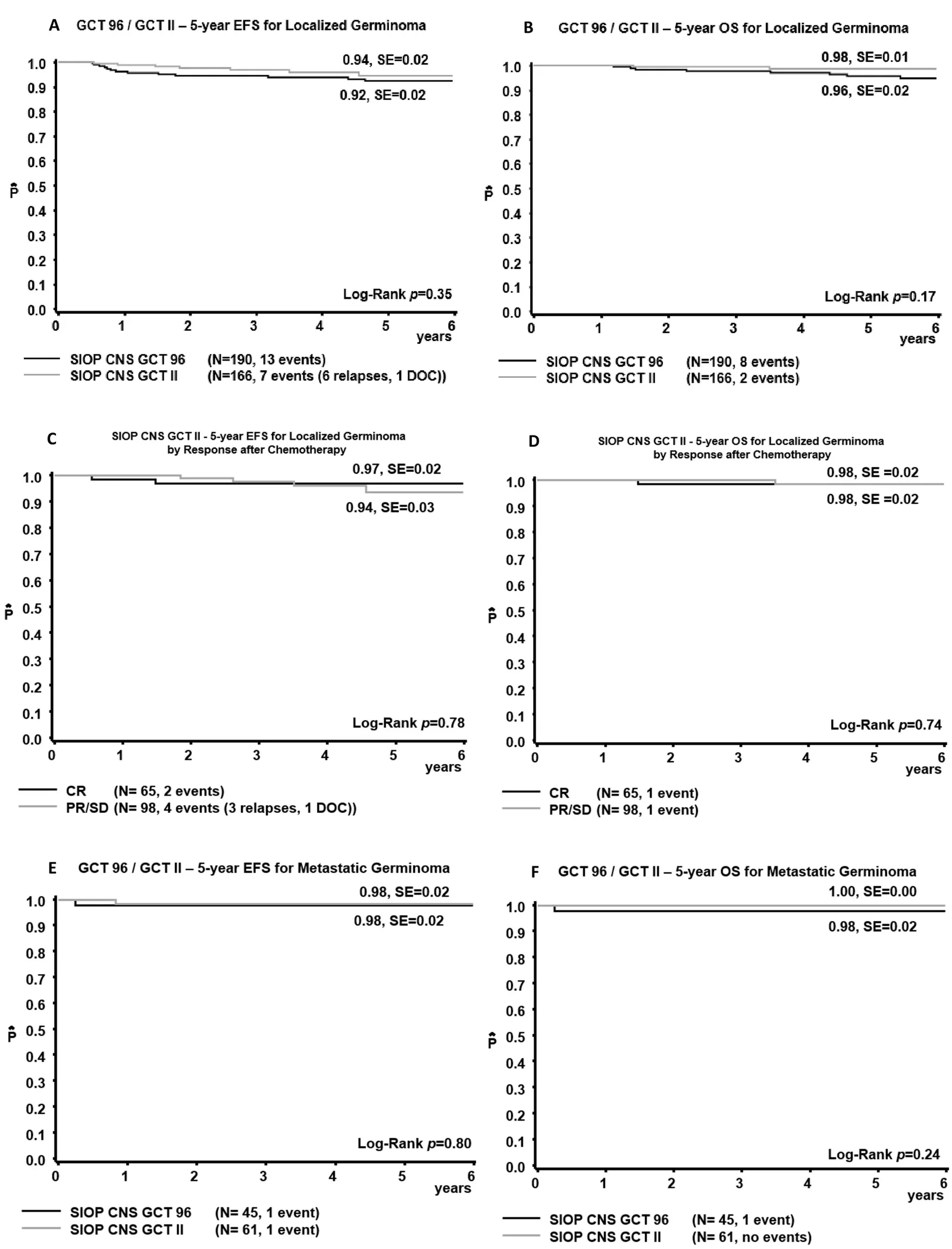
Event-free (EFS) and overall (OS) survival for different groups of patients with intracranial germinoma on the SIOP-CNS-GCT-II trial. CR: complete remission; PR: partial remission; SD: stable disease; EFS: event-free survival; SE: standard error.

Five-year EFS for patients with localized germinoma was 0.94 ± 0.02, similar to results obtained in the previous SIOP-CNS-GCT-96 trial (0.92 ± 0.02; *P *= .35) (Figure 4A). There were 7 events in 166 patients in SIOP-CNS-GCT-II (4.8%), comprising 6 relapses and 1 unknown death of other (non-tumor-related) cause (died of complications; DOC), compared with 13/190 (6.8%) of patients within the SIOP-CNS-GCT-96 protocol, of whom 11 suffered a relapse and 2 DOC (Figure 4A). Five-year OS was 0.98 ± 0.01 for the patients with localized germinoma compared with 0.96 ± 0.02 in the previous SIOP-CNS-GCT-96 trial (Figure 4B). The 2 patients who had germinoma and an immature teratoma component were in CR after chemotherapy and radiotherapy, and neither relapsed.

At data lock, 130/166 (78.3%) patients were in CR, and 2 died. Of the 2 deaths, one was tumor-related (died of disease; DOD) and one was DOC. Thus, in all 166 patients, the response to carbo-PEI induction chemotherapy could be assessed. Sixty-five of 166 (39.2%) were in CR after chemotherapy; 2 of which (2/65) relapsed, 1 locally and 1 combined, 0.5-1.4 y from diagnosis (Figure 2B). Of 90/166 (54.2%) patients in PR after chemo, 88 received 24 Gy WVRT + 16 Gy boost as per trial protocol, of which 2/88 relapsed (1 local and 1 combined), 1.8-2.6 y from diagnosis (Figure 2B). The remaining 2 PR patients received 24 Gy WVRT and focal 40 Gy, with the patient who received WVRT experiencing a relapse. The DOC patient was in PR after chemotherapy. Of the remaining 11/166 evaluable patients, 8 had SD after chemotherapy; second look surgery was not performed in all 8 SD patients. Only 2/8 patients underwent a second-look surgery. Histology from these surgeries revealed that one of these 2 patients had a germinoma with a mature teratoma component, while the other patient only had a mature teratoma. Seven out of 8 SD patients received 24 Gy WVRT + 16 Gy boost, and 1 24 Gy WVRT + 30 Gy boost (presence of teratoma) without relapse. Three patients had PD after chemotherapy; 2 received 24 Gy WVRT [one with 30 Gy boost (for teratoma), 1 without boost], 1 received 24 Gy CSRT + 30 Gy boost. The 2 patients that received WVRT were in PR and CR after radiotherapy, while the patient that received CSRT was in PR after RT and relapsed within 12 months (Figure 2B).

Regarding relapse events, 6/166 (4.2%) of patients with localized disease relapsed during the first 55 months from diagnosis, with a median time of 19 months. Three of 6 (50%) relapses in this group were noted to be bifocal tumors. Of the 6, 2 were in CR after chemotherapy, 3 were in PR, and 1 had PD (Table 1). At the end of the trial, the patient in CR, who received 24 Gy WVRT without boost (patient 6176), remained in CR, and 1 patient from the PR group experienced CR (Table 1). The DOD patient was in CR after chemotherapy and radiotherapy (24 Gy WVRT). The patient died 3 months after experiencing a second relapse, 8 months after the first relapse. Patient 6233 (Table 1) with bifocal disease did not receive the 16 Gy boost as did the other PR patients; the pineal lesion in this patient was classified as CR post-chemotherapy, and the suprasellar lesion was only minimal in size. Therefore, the patient was classified overall as PR, but did not receive a tumor bed boost, as recommended by the reference radiologist and radiation oncologist.

To determine the impact of the risk-adapted approach for localized germinoma, the 5-year EFS for both the CR (24 Gy WVRT alone) and PR/SD (additional tumor bed-boost) response groups after chemotherapy were compared. This demonstrated a 5-year EFS of 0.97 ± 0.02 for the CR group (*n* = 65) compared with 0.94 ± 0.03 for the combined PR/SD group (n = 98) (*P *= .78) (Figure 4C). Two events occurred in the CR group and 4 in the combined PR/SD group. Five-year OS was 0.98 ± 0.02 in the CR group and 0.98 ± 0.02 in the PR/SD group (Figure 4D).

### Metastatic Germinoma

Five-year EFS in patients with metastatic germinoma was 0.98 ± 0.02, identical to that in SIOP-CNS-GCT-96 (*P *= .75), comprising 1 event in each trial: 1/61 (1.6%) in SIOP-CNS-GCT-II and 1/45 (2.2%) in SIOP-CNS-GCT-96 (Figure 4E). Five-year OS was 1.00 ± 0.00 compared with 0.98 ± 0.02 in the previous SIOP-CNS-GCT-96 trial (Figure 4F). Of these 61, after 24 Gy CSRT + 16 Gy boost, 55 (90.2%) patients were in CR and 5 (8.2%) in PR. Only 1/61 (1.6%) of the patients with metastatic germinoma relapsed (at 9 months following diagnosis), after being in CR after radiotherapy (Table 1).

### Acute Toxicity Reporting

Twenty-four CTCAE v3.0 grade 3 and 4 toxicities were reported up to 2 years from trial closure in 17 different patients with germinoma. These comprised 12 serious adverse events (SAEs), 12 serious adverse reactions (SARs), and no unexpected serious adverse reactions (SUSARs). These 24 reports included febrile neutropenia and hematotoxicity (*n* = 4), infections/sepsis (*n* = 3), electrolyte disturbances, other metabolic and nutritional disorders (*n* = 7), nervous system disorders, such as seizures and/or ifosfamide encephalopathy (*n* = 4), and others (*n* = 6). From a radiotherapy perspective, there were no unexpected toxicities, with reported toxicities mainly relating to hematotoxicity, with some reports of headache, nausea, and vomiting. Late toxicities are not reported here.

## Discussion

One of the primary objectives of the SIOP-CNS-GCT-II trial, for patients with germinoma, was to demonstrate that the EFS, using a risk-adapted approach and avoiding CSRT for patients with localized disease, was comparable with the excellent outcomes from the previous SIOP-CNS-GCT-96 study.^2^ Our data confirm the ongoing efficacy of this approach, and further, that the omission of a tumor bed-boost for patients with localized disease in CR following carbo-PEI induction chemotherapy was safe, with no increased risk of events. This defines a new standard-of-care for European patients, with trial data showing the efficacy of dropping the boost for those in CR after chemotherapy for localized (pineal/suprasellar/bifocal) germinoma. Such patients can be treated effectively with a combined treatment approach using carboplatin-based chemotherapy followed by 24 Gy WVRT. Only in cases of residual disease following chemotherapy was an additional tumor boost required.^7^^,^^9^^,^^25-27^ For all such cases, a 16 Gy boost was used, except for those with SD undergoing incomplete resection where the pathology of the resection specimen showed a teratoma component. In such cases, a 30.4 Gy boost was delivered. Although residual disease is an adverse prognostic risk factor for most malignant pediatric brain tumors, neither the presence nor the size of residual tumor was identified as an adverse prognostic factor in the SIOP-CNS-GCT-96 trial.^2^ From a toxicity perspective, induction chemotherapy was well tolerated with no unexpected issues reported.^28^ That notwithstanding, future work will involve attempts to reduce the overall toxicity of chemotherapy by decreasing the use of ifosfamide in such patients. For patients with metastatic germinoma, the SIOP-CNS-GCT-II trial confirmed that 24 Gy CSRT plus a 16 Gy boost to the primary and macroscopic metastatic sites remains the mainstay of treatment.^26^ In future trials, risk-adapted reduction of radiotherapy in both localized and metastatic germinomas would also contribute to the reduction of overall treatment toxicity and long-term effects.^15^ For patients with metastatic germinoma, consideration of chemotherapy use may also facilitate future radiotherapy dose reductions, aiming to further optimize the outcomes for patients with CNS germinoma.^1^^,^^29^

From a radiotherapy perspective, the North American Children’s Oncology Group (COG) assessed dose reductions to 18 Gy + 12 Gy tumor bed-boost (30 Gy total dose) in localized germinoma with CR after chemotherapy, whereas germinoma in PR received 24 Gy + 12 Gy boost in the ACNS1123 trial.^26^ Although the study failed to demonstrate non-inferiority of the 18 Gy WVRT regimen, it showed excellent outcomes, with 3-year progression-free survival of 0.945 ± 0.03 and 0.938 ± 0.06 for the 18 Gy and 24 Gy WVRT cohorts, respectively.^26^  Table 2 summarizes the key differences between the SIOP-CNS-GCT-II and COG ACNS1123 trials from eg, a tumor marker and imaging response assessment perspective.^30^^,^^31^ Work on a SIOP-CNS-GCT-III protocol is underway and will seek to further reduce the late effects of treatment through consideration of further chemotherapy and/or radiotherapy reductions for patients with localized disease. This is important, as delivery of carbo-PEI chemotherapy with concomitant hyperhydration can be challenging, as it is known that ∼50% of such patients have pre-existing DI.^25^ A 1990s study highlighted the efficacy of single-agent carboplatin prior to CSRT in this cohort^7^ and more recently, treatment of a small cohort during COVID with vinblastine prior to radiotherapy has been reported.^25^ As a result, in the UK, the “MonoGerm” trial, opening in 2026, will evaluate carboplatin or vinblastine monotherapy prior to radiotherapy^32^ and compare CR rates after chemotherapy with those observed for carbo-PEI on this trial. Overall, such approaches will aim to continue to maintain excellent survival rates whilst seeking to reduce the overall treatment burden for patients.

**Table 2.** Comparison of the SIOP-CNS-GCT-II and COG ACNS1123 trial-specific information for germinoma patients

	**SIOP CNS GCT II germinoma—Europe** ^30^	**COG ACNS1123 germinoma—USA** ^31^
Tumor marker threshold	AFP <25 ng/ml and ß-HCG <50 IU/l in serum and CSF	AFP ≤10 ng/ml in serum and CSF. ß-HCG 5 - ≤ 50 IU/l in serum and/or CSF. For bifocal, pineal lesion+diabetes insipidus or histologically confirmed germinoma ß-HCG can be ≤100 IU/l in serum and/or CSF; AFP still has to be ≤10 ng/ml in Serum and CSF.
Inclusion criteria	Primary diagnosis of an intracranial germ cell tumor Main residence in one of the participating countries Written consent for trial participation, diagnosis, and treatment according to the protocol, and consent for data transfer	Age between 3 and 21 years at time of enrollment Newly diagnosed with localized primary CNS germinoma Cranial MRI with and without gadolinium at diagnosis/prior to enrollment Lumbar CSF must be obtained prior to study enrollment unless medically contraindicated CSF tumor markers obtained prior to enrollment, unless medically contraindicated Enrolled in ALTE07C1 prior to enrollment in ACNS1123
Exclusion criteria	Primary diagnosis predating the opening of SIOP CNS GCT II Patients with CNS GCTs as second malignancies Patients with a medical, psychiatric, or social condition incompatible with protocol treatment Participation in a different trial for treatment of germ cell tumors and/or concurrent treatment within any other clinical trial. The only exceptions to this are trials with different endpoints, involving aspects of supportive treatment, which can run parallel to SIOP CNS GCT II without influencing the outcome of this trial Pregnancy and lactation Any treatment not given according to protocol prior to registration	Mature teratom or completely resected immature teratoma patients with normal tumormarkers Tumor located outside the ventricles Any prior tumor-directed therapy other than surgical interventation and corticosteroids Pregnancy Lactation, unless the patient ceases breast-feeding Sexually active patients of reproductive potential who have not agreed to use effective contraception for the duration of the study
Stratification	**Non-metastatic**: negative spinal imaging and CSF cytology **Metastatic**: positive spinal imaging and/or CSF cytology	No further stratification pre-chemotherapy (the study was aimed at localized germinoma patients)
Chemotherapy	**Non metastatic**: 2× alternating cycle of carboplatin & etoposide, etoposide & ifosfamide (4 courses total) **Metastatic**: none	**Localized**: 4× courses of carboplatin & etoposide
Radiotherapy	**Non-metastatic**: CR: 24 Gy WVRT PR: 24 Gy WVRT + 16 Gy boost SD: 24 Gy WVRT + 16 Gy boost OR 24 Gy WVRT + 30,4 Gy boost **Metastatic**: 24 Gy CSI + 16 Gy boost	**Localized**: CR: 18 Gy WVI + 12 Gy boost to primary site PR or SD: 24 Gy WVI + 12 Gy boost to primary site
Imaging criteria for treatment response assessment	**CR**: no evidence of disease based on clinical and radiographic assessment. As the pituitary stalk is a structure that physiologically shows contrast enhancement, any kind of abnormal thickening or enhancement has to be categorized as questionable and therefore as PR. Similarly, if anything more than physiological enhancement due to the internal cerebral veins is seen at the pineal gland, the response has to be classified as PR for the purposes of the study and treatment. **PR**: >50% decrease in the sum of the volume of all measurable lesions (calculated from the maximum diameters on MRI in 3 dimensions), no evidence of progression of any lesion, and no new lesions **SD**: <50% decrease in the sum of the product of the volume of all measurable lesions, no evidence of progression of any lesions, and no new lesions **PD**: >25% increase in the size of any measurable lesion and/or appearance of new lesions	**CR**: complete disappearance of visible disease on imaging allowing for iminimal residual disease/enhancement ≤ 0.5 cm maximal dimension in suprasellar or ≤ 1 cm in pineal locations. **PR**: > 0.5 cm dimension residual in the suprasellar area or >1 cm residual in case of pineal involvement after completion of chemotherapy, but ≥65% decrease in the sum of the products of the 3 perpendicular diameters (volume) of all target lesions (up to 5), taking as reference the initial baseline measurements **SD**: Neither sufficient decrease in the sum of the products of the 3 perpendicular diameters of all target lesions to qualify for PR (taking as reference the initial baseline measurements), nor sufficient increase in a single target lesion to qualify for PD (taking as reference the smallest disease measurement since the treatment started), and residual disease after chemotherapy of >1.5 cm maximal diameter. **PD**: 40% or more increase in the product of perpendicular diameters (volume) of ANY target lesion, taking as reference the smallest product observed since the start of treatment, or the appearance of 1 or more new lesions.

Trial-specific information on the tumor marker thresholds, inclusion and exclusion criteria, stratification, chemotherapy, radiotherapy, and imaging criteria for treatment response assessment for germinoma patients are listed.

In the SIOP-CNS-GCT-II study, bifocal cases did not need histological confirmation if patients had a typical presentation, tumor markers below European thresholds, and macroscopic disease at both pineal and suprasellar sites, in which case they were assigned as germinoma. They were considered localized if they had no other non-contiguous disease elsewhere and negative CSF cytology. In a recent study where histological verification of patients presenting with bifocal lesions, DI, and supposed negative tumor markers was performed, 3 of 89 cases (3.4%) were NGGCT.^33^ Of note, on careful re-review, none of these 3 NGGCT cases actually had a full set of tumor markers performed (analysis of both AFP and HCG in the serum and CSF).^33^ Conversely, no other lesions other than germinoma were identified in patients presenting with complete sets of tumor markers in both compartments below specified thresholds.^33^ Whilst recognizing that a very occasional NGGCT case can occur in such bifocal cases, this work underscores the appropriateness of the European approach to bifocal disease, avoiding a large number of unnecessary diagnostic biopsies. Of note, in future European studies, the bifocal definition will be updated to include patients with pineal tumor and DI who do not have macroscopic suprasellar disease on imaging, in line with the North-American and European consensus on imaging of CNS GCT.^34^

In the era of SIOP-CNS-GCT-II, COG, and the European group used different criteria for assessing radiological response to treatment for patients with CNS germinoma.^34^ The more conservative European criteria have resulted in a higher proportion of patients being classified as PR, some of whom would have been classified as CR using the more permissive COG ACNS1123 criteria.^31^ This makes comparison of different retrospective trials and their study outcomes challenging. To overcome this, a consensus paper for imaging response assessments for patients with CNS GCTs was published.^34^ This will serve in the forthcoming SIOP-CNS-GCT-III and COG trial as the new standard and will facilitate retrospective study comparisons as well as further international cooperation in this rare disease type.

Limitations of the SIOP-CNS-GCT-II trial include that the type of radiotherapy was not consistently recorded; we do not, therefore know in all cases which patients received proton vs photon therapy. The SIOP-CNS-GCT-III trial will include centralized quality control for radiotherapy, which will mandate such reporting. Additionally, the lack of patients with basal ganglia germinoma hampers our understanding of the full spectrum of CNS germinoma disease. As a result, we will continue to rely on data from Asian studies to inform us on this entity. Further, review of trial data for bifocal disease has highlighted varying approaches taken by radiation oncologists regarding the delivery of the boost to pineal and suprasellar areas in patients with bifocal disease,^33^ which will be a focus for future work. At present, we continue to advise caution in dropping boosts to both bifocal sites of disease if only one is in CR and the other in PR. Moreover, the lack of long-term cognitive follow-up data makes it difficult to determine whether the RT dose reduction has actually resulted in a substantial decrease in long-term neurocognitive side-effects. Finally, from a biological perspective, given the development of non-invasive biomarkers for malignant CNS GCTs since the SIOP-CNS-GCT-II trial was written, including microRNAs^35-37^ and circulating tumor DNA,^38^ formal biological endpoints will be embedded in SIOP-CNS-GCT-III alongside QoL and neurocognitive secondary objectives.

## Conclusion

In summary, the SIOP-CNS-GCT-II trial has demonstrated the safety and efficacy of dropping the 16 Gy radiotherapy boost for patients with localized (pineal/suprasellar/bifocal) germinoma who achieved CR after induction chemotherapy, thus being cured with chemotherapy and 24 Gy WVRT alone. As EFS and OS in patients with localized or metastatic germinoma are excellent, the results of the trial will facilitate future considerations of chemotherapy and/or radiotherapy dose reductions, aiming to optimize the outcomes for this patient cohort.

## Data Availability

Individuals who wish to access the anonymized trial data can e-mail the SIOP-CNS-GCT-II trial coordinator, Dr. Gabriele Calaminus, stating which study data they wish to view and the rationale. This request will then be reviewed by the international SIOP-CNS-GCT-II trial committee.
